# Usefulness of a Sphincterotome Blade for Removing a Bile Duct Stone Displaced Into an Intrahepatic Duct

**DOI:** 10.1111/den.70291

**Published:** 2026-09-18

**Authors:** Akinori Maruta, Shinya Uemura, Masahito Shimizu

**Affiliations:** ^1^ First Department of Internal Medicine Gifu University Hospital Gifu Japan

**Keywords:** endoscopic retrograde cholangiopancreatography, endoscopic sphincterotomy with balloon dilation, migrated common bile duct stone, sphincterotome blade

**Abbreviations**
CBDS: common bile duct stone
ERCP: endoscopic retrograde cholangiopancreatography
ESBD: endoscopic sphincterotomy with balloon dilation
IHBD: intrahepatic bile duct

## 1 Brief Explanation

Endoscopic retrograde cholangiopancreatography (ERCP) is the standard treatment for common bile duct stones (CBDSs), and balloon or basket catheters are useful for removal of CBDSs [1, 2, 3]. However, CBDSs can sometimes displace into the intrahepatic bile duct (IHBD), making stone removal challenging even with these devices. Here, we report a case in which we successfully removed a CBDS that had displaced into the IHBD using a sphincterotome blade.

**FIGURE 1:**
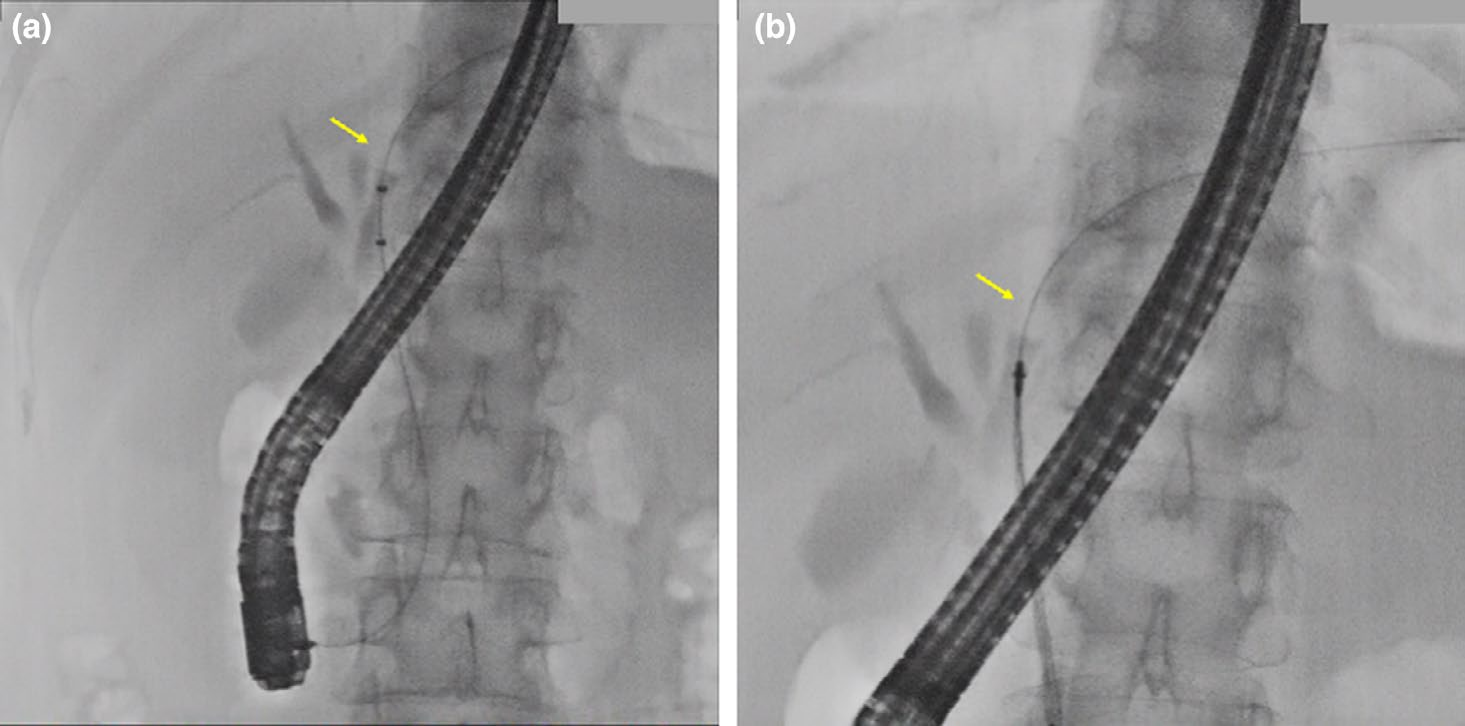
Migrated CBDS (yellow arrow) into intrahepatic bile ducts using stone removal devices. (a) Balloon catheter, (b) Basket catheter.

**FIGURE 2:**
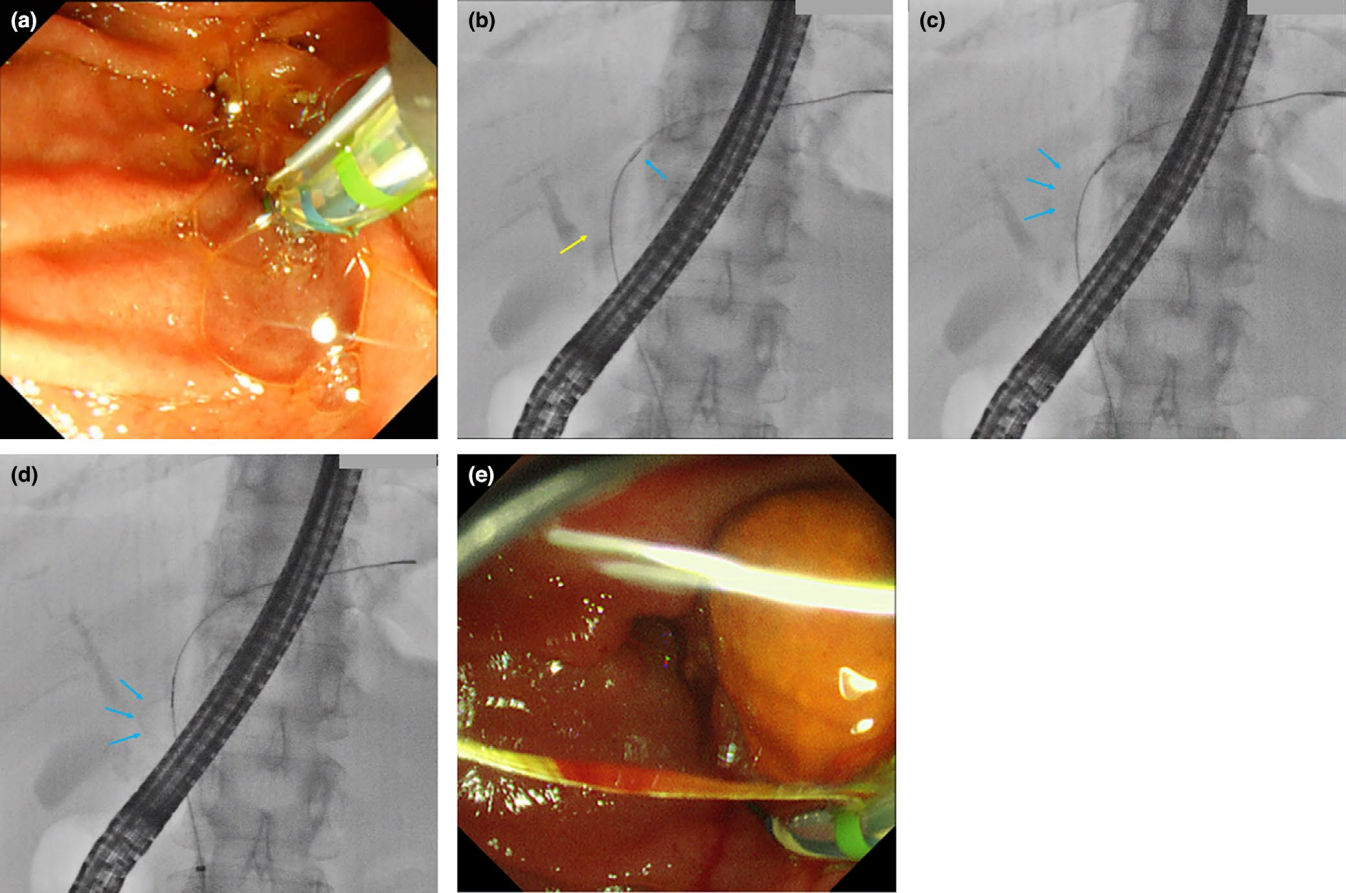
(a) Sphincterotome was inserted into the bile duct. (b) Tip of the sphincterotome (blue arrow) could pass through the displaced stone (yellow arrow). (c) Sphincterotome blade was extended (blue arrows). (d) Displaced stone was grasped with a sphincterotome blade (blue arrows). (e) Migrated stone was successfully removed by grasping it with a sphincterotome blade.

A 59‐year‐old woman presented with abdominal pain, and various imaging examinations revealed CBDSs. ERCP was performed to remove the stones (Video 1). After endoscopic sphincterotomy with balloon dilation (ESBD) using a dedicated device combining a sphincterotome and a papillary dilation balloon (StoneMaster V; Olympus, Tokyo, Japan), a balloon catheter was used to remove the CBDS. When the balloon catheter was advanced to remove the remaining stone at the hepatic hilum, it could not pass through the stone, and the stone moved further into the peripheral IHBD. The basket catheter also failed to pass through the stone and only pushed it into the IHBD (Figure 1a,b). Therefore, the same sphincterotome used for the papillary intervention was inserted into the bile duct and advanced carefully so that its tip passed the stone without pushing the stone into the IHBD. The sphincterotome blade was extended, and the stone was grasped with the extended cutting wire under fluoroscopic guidance. The stone was successfully removed by carefully withdrawing the catheter while holding the stone in place (Figure 2a–e).

**VIDEO 1:** Removal of a bile duct stone displaced into an intrahepatic duct using a sphincterotome blade. Video content can be viewed at https://onlinelibrary.wiley.com/doi/10.1111/den.70291.

Although some reports indicate that cholangioscopy‐guided electrohydraulic or laser lithotripsy is effective for IHBD stones [4, 5], there is currently no established treatment strategy for CBDSs that have displaced into the IHBD. For IHBD stones that are difficult to remove using a conventional balloon or basket catheter, a sphincterotome blade may be considered as a rescue option in selected cases.

## Author Contributions

Akinori Maruta wrote the manuscript. Akinori Maruta, Shinya Uemura, and Masahito Shimizu managed the patients. Akinori Maruta took the correspondence. All authors read and approved the final manuscript.

## Funding

The authors have nothing to report.

## Conflicts of Interest

The authors declare no conflicts of interest.

## Data Availability

Data sharing not applicable to this article as no datasets were generated or analysed during the current study.
